# Design and validation of a self-administered test to assess bullying (bull-M) in high school Mexicans: a pilot study

**DOI:** 10.1186/1471-2458-13-334

**Published:** 2013-04-11

**Authors:** Arnulfo Ramos-Jimenez, Abraham Wall-Medrano, Oscar Esparza-Del Villar, Rosa P Hernández-Torres

**Affiliations:** 1 Instituto de Ciencias Biomédicas, Universidad Autónoma de Ciudad Juárez, Av. Plutarco Elías Calles y Hermanos Escobar s/n, Ciudad Juárez, Chihuahua, Mexico; 2 Escuela de Educación Física y Ciencias del Deporte, Universidad Autónoma de Chihuahua, Chihuahua, Mexico

**Keywords:** School violence, Bullying, Mexico, Validation

## Abstract

**Background:**

Bullying (Bull) is a public health problem worldwide, and Mexico is not exempt. However, its epidemiology and early detection in our country is limited, in part, by the lack of validated tests to ensure the respondents’ anonymity. The aim of this study was to validate a self-administered test (Bull-M) for assessing Bull among high-school Mexicans.

**Methods:**

Experts and school teachers from highly violent areas of Ciudad Juarez (Chihuahua, México), reported common Bull behaviors. Then, a 10-item test was developed based on twelve of these behaviors; the students’ and peers’ participation in Bull acts and in some somatic consequences in Bull victims with a 5-point Likert frequency scale. Validation criteria were: content (CV, judges); reliability [Cronbach’s alpha (CA), test-retest (spearman correlation, r_s_)]; construct [principal component (PCA), confirmatory factor (CFA), goodness-of-fit (GF) analysis]; and convergent (Bull-M vs. Bull-S test) validity.

**Results:**

Bull-M showed good reliability (CA = 0.75, r_s_ = 0.91; p < 0.001). Two factors were identified (PCA) and confirmed (CFA): “bullying me (victim)” and “bullying others (aggressor)”. GF indices were: Root mean square error of approximation (0.031), GF index (0.97), and normalized fit index (0.92). Bull-M was as good as Bull-S for measuring Bull prevalence.

**Conclusions:**

Bull-M has a good reliability and convergent validity and a bi-modal factor structure for detecting Bull victims and aggressors; however, its external validity and sensitivity should be analyzed on a wider and different population.

## Background

Bullying at schools is defined as systematic physical, verbal and/or psychological abuse from one or more students toward another [1]. Involved parties are identified as aggressor(s) (“bully”) and victim (“bullied”), respectively. The prevalence of this psychopathology is very high around the globe ranging from 9% to 45% and 5% to 36% among boys and girls, respectively [2,3]. In Mexico, it goes from 10% to 83%, with those living in highly violent, poor, and insecure US-border cities being particularly vulnerable [4-7]. However, despite these figures and being considered a psychiatric disorder [8,9], its detection and management is often restricted to school and social services.

Bullying implies the presence of certain psychopathological behaviors in aggressors and victims. Manifestations in the former include threatening, harassment, mocking, menacing, discrediting, or insulting [8], which requires timely psychiatric attention [9]. The victims generate many health disturbances, anxiety and depression [10-12], and problems with interpersonal relationships [13,14]. In turn, these behaviors may result in domestic violence, criminality, substance abuse [15], and even suicidal thoughts [16]. There are significant differences in such bullying manifestations according to gender and socioeconomic status [17]; although other social factors also contribute such as having a family that promotes violence, teachers that ignore or dismiss bullying, schools that have a negative social climate, or students who socialize with bullies (aggressors) [18].

Several screening methods for detecting bullying have been applied within and outside of the school environment. Qualitative observation methods have been used successfully to evaluate students’ behavior at school, which constitute the most common assessment strategy used by school professionals [19]. Direct interviews and self-reports have also been used to establish the incidence of bullying, its impact on student development, and the effectiveness of anti-bullying interventions [20,21]. However, besides being time-consuming, these methods do not measure the true prevalence of bullying due to the fact that they rely on anonymity [22], so victims are overestimated while aggressors are underestimated [23]. Recently, detection of adolescent victimization through mobile phone and internet tests have been proposed to guarantee anonymity [24]. Although reliable for detecting self-perceived stress, loneliness, traditional victimization, and satisfaction with life, these tests have been applied only to asses cyber bullying and may prove to be very sophisticated for poor populations that do not have access to these technologies.

Validated, self-administered tests are practical and economic methods for evaluating bullying behaviors. They can be grouped into two categories: a) Those that allow analysis of the incidence anonymously and b) those that seek to evaluate a particular aspect of the problem through custom surveys or projective situations [25,26]. In Mexico, the type and incidence of students’ aggressive behaviors have been studied by using the Bull-S test [9,26] and the Concept of Intimidation among Equals (CIMEI) test [8]. The former is a self-administered 15-item questionnaire for collective application and was developed mainly to detect aggressiveness among peers within the school context from a double perspective: students and teachers. It attempts, in a 25 to 30 minute administration, to identify aggressors, victims, victim-aggressors, and neutral parties. The problem with Bull-S, as occurs with other tests [26], is that it asks for personal information from the respondent, which does not guarantee anonymity and reduces the answering veracity. CIMEI detects harassment or mistreatment at school and includes three sections, each of them aimed at a different audience: Students, teachers, and parents. The student section includes 12 questions with multiple-choice, descriptive answers; it can be self-applied; and it has good internal consistency. Although the structure of this test allows the examiner to classify bullying into three different roles (victim, aggressor, and victim-aggressor), the operational definition of these roles within the test is somewhat difficult and confusing [9,26].

The aim of this study was to validate a 10-item self-administered test (Bull-M) for assessing bullying among high school Mexicans. The development of a test with fewer questions (as compared to Bull-S) and whose only intention is to rapidly characterize the behavior of victims and aggressors may help to build epidemiological indicators without sacrificing the anonymity of the respondent.

## Methods

### Design and structure of bull-M

The test was originally designed by four experts in the fields of social sciences and community health (ARJ, AWM, OEDV and RPHT). They were supported by seven teachers with over five years of experience in bullying (Bull) management at schools with a high incidence of violence and drug abuse in Ciudad Juarez (Chihuahua, México). Focus groups and in-depth interviews were conducted to identify the most common Bull behaviors observed in or out of school. Ten questions (items) were included in the final test (Additional files 1 and 2): Five (items 1 to 5) about twelve Bull behaviors (representations) occurring in or out of school, four (items 6 to 9) on the student’s and/or peer’s participation in bullying acts, and one (item 10) on several somatic consequences in bullying victims (“*In the last four weeks, how often have you had a stomachache, headache, loss of appetite, or problems sleeping?”*) [12,26,27]. Also, in order to evaluate the frequency of each experienced situation or the student involvement in them, a 5-point Likert scale was added: never, rarely, sometimes, often, and every day. Only for validation purposes, this frequency was coded on a scale of 0–5. Lastly, although designed and applied in Spanish (Additional file 1), an English version of Bull-M is also provided (Additional file 2).

A preliminary version (9 items) of Bull-M, in which the order of all items was not that of the final instrument, was administered to 20 students (13–15 years old), to make sure that it was understandable and to evaluate response time. Although all participants found it clear, a certain degree of intimidation was observed due to the order of items. It was then decided to begin with another question (*How often do your classmates allow or invite you to participate in their games, school activities, or extracurricular activities?*) as the initial item (Additional files 1 and 2). Before answering the test, the general instructions were explained individually as well as the anonymous nature of Bull-M in order to reinforce the students’ confidence. The test was efficiently applied in 10–15 minutes by two collaborators (ARJ, OEDV) collectively within the classroom while the teacher was not present.

#### Survey

Bull-M was applied from February to May of 2011 among 400 students (60% male, 13.4 ± 1.1 y) from three out of 25 high schools located on the outskirts of Ciudad Juarez, zones with a long history of violence and poverty (bullying elicitors). The sample, although sampled completely randomly, was not representative of the population at all high schools in Ciudad Juarez. Two hundred participants were further selected for a second re-examination (test-retest validity) 14 days after the first application. An informed consent for participation was obtained from each parent, from school authorities, and from each participant. The protocol was approved by the Ethics Committee of the Autonomous University of Chihuahua (UACH).

#### Validation

Four criteria were chosen to validate Bull-M: a) content (CV, judges), b) reliability [Cronbach’s alpha (CA) and test-retest], c) construct [principal component (PCA) and confirmatory factor (CFA) analysis and goodness of fit (GF)], and d) convergent validity (Bull-M vs Bull-S). Their characteristics, rationale, procedure, and statistics used are described below:

#### Content validity (CV)

In the social sciences, CV (also known as *logical validity*) is important for demonstrating the degree to which a written test fulfills its purpose. Generally, this is the first validation criterion because it examines the overall comprehension of the test. It is usually performed by experts on the studied phenomenon (judge validation) who evaluate the design characteristics of the test as well as each individual item. In this study, CV was performed by twelve judges (RPHT, AWM, and ten more) by using the Expert Judgment Validity Test (EJVT; Additional file 3). All items (n = 19) included in EJVT were designed to intentionally evaluate the following characteristics of Bull-M: content, size, order, accuracy, and answering format, by using a 3-point Likert scale, which were further scored as follows: Poorly (0), fairly (1), and sufficiently (2). The average score (AS) for each item was then calculated (mean ± SD). An AS <1.5 to any item was taken into account for redesign when needed.

#### Reliability

This type of validation is required for “adjusting” a test and contributes to its “most convenient” format. There are many statistical indicators used for this type of validation but the two most common are Cronbach’s alpha, which evaluates the consistency of results across items within a test, and test-retest, which evaluates the degree to which the test scores are consistent from one test application to the next. Here, a Cronbach’s alpha from 0.70-0.80, 0.81-0.90 and ≥0.90 was considered as acceptable, good, and excellent, respectively [28]. Test-retest reliability was evaluated in 200 students within a 14 day frame. The difference between the items score of the first (test) and second application (retest) was evaluated by Spearman correlation (r_s_).

#### Construct validity

This was performed by principal component analysis (PCA) and confirmatory factor analysis (CFA), the latter through a structural equation model. PCA has two objectives: a) to reduce the number of items in a written test while retaining the variability of the data and b) to identify hidden patterns (components or factors) to classify them according to their contribution to the final test score. PCA is generally followed by CFA whose main objective is to test whether all items fit a hypothesized measurement model.

PCA and CFA were performed with samples of 200 and 198 participants, respectively. The factor structure of PCA was ascertained by Varimax rotation following the Kaiser-Guttman criterion in which eigenvalues are taken ≥1.0 as a decision rule [29]. Also, in order to ensure an adequate representation of the variables, only those items whose communality (proportion of their variance explained by the factor) was ≥0.45 were included. Finally, in order to evaluate the fitting of the sampling and the possible sphericity of the data collected, the Kaiser-Meyer-Olkin (KMO) [30] and Bartlett [31] tests were applied. Lastly, the following goodness of fit indicators were calculated: Root mean square error of approximation (RMSEA), goodness of fit index (GFI), adjusted goodness of fit index (AGFI), comparative fit index (CGI), and normalized fit index (NFI). The model of structural equations was analyzed with Amos 16.0 (Amos development corporation, USA) while other analyses were done with PASW Statistic 18.0.

#### Convergent validity

This refers to the degree to which two measures of constructs (e.g. two tests) that theoretically should be related are in fact related. Here, 100 participants from the second application (retest) of Bull-M were also invited to answer Bull-S. Bull-S is mainly used to detect Bull roles (victim, aggressor, and victim-aggressor) [9,26]. However, one of its items (item #13) is related to the frequency in which Bull acts occur (*how often the aggressions occur?*) on a 4-point Likert scale (every day, twice a week, rarely, and never) [32]. Pooled frequencies (%) from the “bullying other” subscale (items 6–9) of Bull-M and from item #13 of Bull-S were then compared, making a proper adjustment of both frequency scales for a proper comparison [Sometimes + often (Bull-M) = Twice a week (Bull-S)].

## Results

### Content validity (CV)

There were no problems with the administration or comprehension of Bull-M. All 12 judges agreed that Bull-M was a valid test for assessing Bull behaviors (EJVT total score = 0.93).

### Reliability

The internal consistency of Bull-M was acceptable (Cronbach’s α = 0.75). Item #1 showed the lowest correlation (r_s_ = 0.10) with the total score of Bull-M, although its elimination did not improve the internal consistency of Bull-M (Table 1). Test-retest evaluation revealed statistical differences (p < 0.05) between pre and post-application (14 days later) of Bull-M for items 3, 5, 7, and 9. However, the correlation of total scores (from all participants) for both applications turn out to be excellent (r_s_ = 0.91; p < 0.001).

**Table 1:** Reliability of Bull-M

**Item**	**Internal consistency**^**1**^		**Reproducibility **^**4**^	
	**r**_**s**_^**2**^	**α**^**3**^	**Test**	**Retest**
1	.10	.79	1.9 ± 0.09	1.9 ± 0.09
2	.48	.72	0.7 ± 0.06	0.8 ± 0.06
3	.40	.73	0.5 ± 0.05	0.9 ± 0.05*
4	.53	.71	0.8 ± 0.06	0.9 ± 0.06
5	.47	.72	0.7 ± 0.06	0.9 ± 0.06*
6	.44	.72	0.9 ± 0.06	1.0 ± 0.06
7	.49	.72	0.4 ± 0.05	0.6 ± 0.05*
8	.52	.71	0.8 ± 0.06	0.9 ± 0.06
9	.55	.71	0.7 ± 0.06	0.9 ± 0.06*
10	.34	.74	1.2 ± 0.08	1.1 ± 0.06

^1^ Cronbach’s α for total test = 0.75; ^2^Item-total correlation; ^3^ Adjusted Cronbach’s α if item is deleted; ^4^mean ± EE; *statistical differences between test (p < 0.05).

### Construct

**Figure 1:**
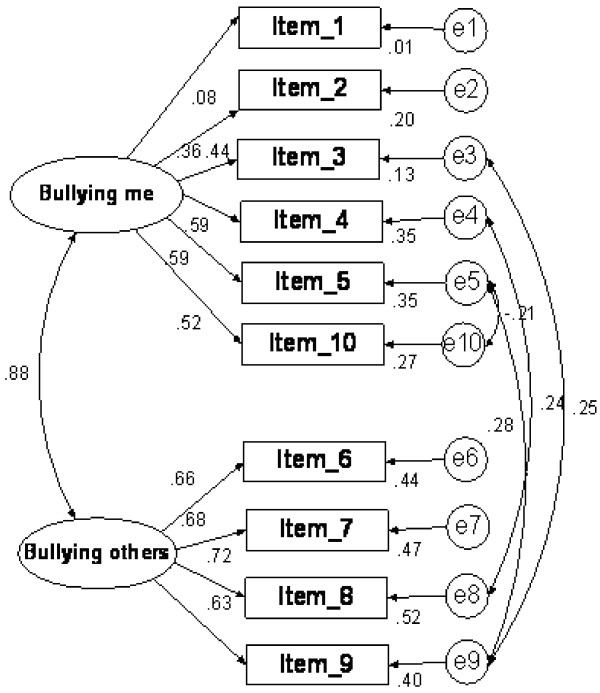
**Confirmatory factor analysis (CFA) of Bull-M test: Standardized estimates; Statistics: *χ***^**2**^ **= 35.8, df = 30, p = 0.21.**

PCA evidenced two factors: “Bullying me” (items 1–5, 10) and “Bullying others” (items 6–9; Table 2). Kaiser-Meyer-Olkin (KMO) sampling adequacy was 0.77 while the Bartlett sphericity test was statistically significant (*χ*^2^ = 404.341; *p* < 0.001). Loading factors for all items were ≥0.49 and the factorial model explained 44.6% of Bull-M variance. CFA not only confirmed the bifactorial structure detected with PCA (*χ*^2^ = 35.8, df = 30, p = 0.21; Figure 1) but also showed a high correlation between these two factors (r = 0.88). Lastly, the model showed an adequate goodness of fit: RMSEA = 0.031, GFI = 0.97, AGFI = 0.94, CGI = 0.99 and NFI = 0.92.

**Table 2:** Construct validity of Bull-M

**Item**	**Variance**^**1**^	**Factor loading **^**2**^	
	**Explained (%)**	**“Bullying other”**	**“Bullying me”**
1	32.88		.596
2	11.69		.638
3	9.99		.569
4	9.78		.608
5	8.47		.493
6	7.94	.730	
7	5.74	.613	
8	5.66	.730	
9	4.21	.537	
10	3.63		.490

^1^ Principal component analysis (PCA) ^2^ Varimax Rotation.

### Convergent validity

Pooled frequencies (%) from the “bullying other” subscale (items 6–9) of Bull-M and from item #13 from Bull-S were similar (Table 3): Never (~3.6%), everyday (~18%) while 78% were distributed among intermediate frequencies.

**Table 3:** Convergent validity of Bull-M

**Bull-M**		**Bull-S**	
Never	3.5%	Never	3.8%
Rarely	17.6%	Rarely	42.9%
Sometimes	31.7%	Twice a week	35.3%
Often	27.6%		
Everyday	19.6%	Everyday	17.1%

In the Bull-M the average of items 6 to 10 were considered, in the Bull-S only the item 13.

## Discussions

Bullying (Bull) at schools in Mexico is reaching epidemic proportions. According to the *First National Survey of Exclusion, Intolerance and Violence in Public Schools*[33], surveyed by the Ministry of Public Education (SEP) in 2008, 44.6% and 26.2% of men and women between 15 and 19 years old recognized having abused their peers. On the other hand, the *National Commission on Human Rights (*CNDH) estimates that 40% of all Mexicans in elementary schools, both public and private, are victims of bullying [34]. Also, previous studies have reported specific cultural conflicts and discrimination between ethnic groups due to differences in acculturation (e.g. language barriers), an additional factor seen in Mexican immigrants [35] and those settled in Mexico-US border cities (like the one studied here) [7]. However, there have been very few systematic studies performed in Mexico on Bull epidemiology, none of them reviewing Bull impact on public health costs or its implications for community health.

Studies in social psychology indicate that Bull aggressors and victims are characterized by displaying recognizable behaviors [36]. However, from an epidemiological standpoint, the psychosocial distress in both parties is not easy to detect because it depends largely on the complicity and anonymity provided by peers. As a consequence, Bull epidemiology and its early detection, at least in Mexico, is limited by the lack of validated tests to ensure the respondents’ anonymity. Here, the preliminary study and experts’ opinions showed that interviewing about school bullying can be intimidating. This can cause serious problems for the correct identification of both aggressors and victims of bullying. In this study it was observed that the addition of a single less intimidating question at the beginning, although having a low correlation with the overall scale (r_s_ = 0.10; Table 1), did not affect the reliability of Bull-M. To our knowledge, there is no previous report that analyzes the intimidating effect of individual questions on the truthfulness of total response of any test that assesses bullying.

On the other hand, validation statistics used in this study (reliability, construct, and convergence) demonstrated that Bull-M is a useful tool for population studies. This assertion is supported by the following:

*First,* the internal consistency (Cronbach’s α; CA) and reproducibility (test-retest) showed that Bull-M is *reliable*. The general consensus on the interpretation of CA stating that α <0.70 means that a test is not uniform while an α > 0.90 suggests the existence of redundant items [34]. Cronbach’s α for Bull-M was 0.75, signifying acceptable consistency. Baldry [37] and Cerezo [26] found similar CA results with Bull-S and a self-report anonymous questionnaire, respectively. Also, a high correlation was found between a first and second application [test-retest (r_s_ = 0.91; p < 0.001)]. Although a higher score (p < 0.05) in retest was observed for items 3, 5, 7, and 9, this is non-meaningful because the transformation of the Likert scale (never, rarely, sometimes, often, and every day) into numbers (0, 1, 2, 3, 4) really shows that at both times, these items were identified as “rarely.” However, it should be kept in mind that the test-retest may be influenced by the complexity and ambiguity of the item [38]; although both the expert panel and the pilot study agreed on the fact that Bull-M is simple and clear, so complexity and ambiguity are not an issue.

*Second*, CPA and CFA correctly identified the bi-factorial character of Bull-M as was proposed from the beginning. CPA revealed that both factors (“Bullying me” and “Bullying others”) explained 44.6% of total variance. Although there are tests which explain up to 75% of total variance [6], it is more common to find lower percentages [37]. A plausible explanation to this is the fact that no single test can contain all possible bullying representations as is the case of Bull-M which only reflect twelve situations (Additional files 1 and 2) which were gathered from in-depth interviews and focus groups with experts and teachers. Also, PCA is not able to demonstrate each factor structure or the dimensionality of the model [39], but CFA does; it confirmed the bi-factorial structure suggested by PCA, providing not only more evidence on the robust structure of Bull-M but also on the strong association between both factors.

*Third*, Bull-M was designed to allow assessment of the prevalence of bullying in schools which it actually allows, at least when compared to Bull-S (convergent validity). To our knowledge, comparisons on the psychometric properties of two tests designed to explore Bull at schools, have not yet been reported.

### Limitations

The authors recognize that there are many validation factors that need to be addressed in order to sustain even more the reliability and criterion validity of Bull-M. *First*, internal consistency and reproducibility, although commonly used in social sciences, are just two of many reliability criteria. External validation conducted on different populations with different social conditions could argue the reliability of Bull-M even more. *Second*, given the inherent subjectivity of any test that attempts to evaluate Bull behaviors, the calibration or criterion validity is difficult to evaluate since there is no “golden” criterion with which to contrast [39]; nevertheless, it has to be analyzed somehow. *Third*, although Bull-M was originally designed to be applied on subjects between the ages of 8 and 15, it also has to be validated with elementary school students because Bull at this age is somewhat different [40].

## Conclusions

In this study, we have reported a suitable tool (Bull-M) for assessing bullying among high school Mexicans that may help to build epidemiological indicators without sacrificing the anonymity of the respondent. Bull-M showed good internal consistency, reproducibility and a robust structure capable of detecting two Bull roles (victim and aggressor). The number of items (n = 10) and factors (two) of Bull-M make it an ideal instrument for population surveys since it is easy to apply, and its results are economic. Moreover, it has the potential ability to evaluate not only the frequency (and thus the prevalence) of Bull situations and roles (aggressor/victim) but also has the ability to prioritize the types of Bull behaviors and their somatic effects on victims (data not reported here).

### Recommendations

This paper is merely the first to demonstrate the potential of Bull-M for assessing Bull at schools. This highlights the need for further external validity not only in multiple populations but also with the inclusion of other criteria validity factors (e.g. perception of parents and teachers).

### Implications for school health

Bull-M can improve the gathering of information on the presence of Bull in high school, allowing the design of better intervention programs to reduce this public health concern.

## Competing interests

The authors declare that they have no competing interests.

## Authors’ contributions

ARJ, AWM, OEDV, and RPHT were responsible for designing Bull-M and for preparing and reviewing of the final manuscript. ARJ and OEDV undertook the survey and the statistical analysis. AWM and RPHT coordinated the panel of judges. All authors read and approved the final manuscript.

## Pre-publication history

The pre-publication history for this paper can be accessed here:

http://www.biomedcentral.com/1471-2458/13/334/prepub

## Supplementary Material

Additional file 1 Cuestionario sobre la presencia de intimidación (Bullying) dentro y fuera de la escuela (Bull-M). Click here for file

Additional file 2 Test on the presence of bullying in and out school (Bull-M). Click here for file

Additional file 3 Expert judgment validity test (EJVT). Click here for file
