# Supplementary material for: Design and validation of a self-administered test to assess bullying (bull-M) in high school Mexicans: a pilot study
Source: BMC Public Health. 2013 Apr 11;13:334. doi: 10.1186/1471-2458-13-334 (PMC3671223; doi:10.1186/1471-2458-13-334)
Supplement: Additional file 1 — Cuestionario sobre la presencia de intimidación (Bullying) dentro y fuera de la escuela (Bull-M). [file 1471-2458-13-334-S1.doc]

Additional file 1

**Cuestionario sobre la presencia de intimidación (Bullying) dentro y fuera de la escuela (Bull-M)**

Este cuestionario es confidencial y la información proporcionada solo será utilizada con fines estadísticos. De esta manera, tu identidad será protegida. Te pedimos que no pongas tu nombre en ninguna parte del documento y que contestes lo más honesto posible a cada pregunta, sobre lo que pasa dentro y fuera de la escuela ya sea por ti, tus compañeros de clase o amigos. Además, marca con una “X” las acciones (en negritas) que más se aplican a ti o a lo que tú haces. Ejemplo:

**
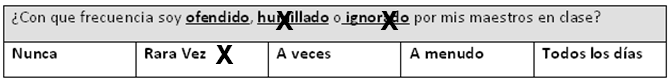
**

Lo anterior significa que rara vez eres humillado o ignorado por tus maestros en clase

| Edad: años Genero: Masculino ( ) Femenino ( ) |
| --- |

**¿Qué pasa dentro o** fuera de la escuela?

| 1. ¿Con que frecuencia tus compañeros permiten o te invitan a participar en sus juegos o actividades intra o para escolares? | | | | |
| --- | --- | --- | --- | --- |
| **Nunca** | **Rara Vez** | **A veces** | **A menudo** | **Todos los días** |
| 1. ¿Con que frecuencia eres **excluido(a)**, **rechazado(a)** o **expulsado(a)** por tus compañeros en juegos, conversaciones, o actividades escolares o para escolares? | | | | |
| **Nunca** | **Rara Vez** | **A veces** | **A menudo** | **Todos los días** |
| 1. ¿Con que frecuencia estas **obligado(a)** o **amenazado(a)** por tus compañeros para hacer cosas que te hacen daño, te ofenden o que tu no deseas hacer? | | | | |
| **Nunca** | **Rara Vez** | **A veces** | **A menudo** | **Todos los días** |
| 1. ¿Con que frecuencia tus compañeros de clase se **burlan** de ti, te **insultan**, te **castigan**, te **golpean**, o te **hacen daño**? | | | | |
| **Nunca** | **Rara Vez** | **A veces** | **A menudo** | **Todos los días** |
| 1. ¿Con que frecuencia eres **culpado** o **acusado** por tus compañeros de clase por alguna ofensa que puedes o no haber cometido tú? | | | | |
| **Nunca** | **Rara Vez** | **A veces** | **A menudo** | **Todos los días** |

**¿Qué hacen tu o tus compañeros de clase dentro o** fuera de la escuela?

| 1. ¿Cuántas veces tu o tus compañeros de clase han **excluido**, **rechazado** o **expulsado** a alguien de juegos, conversaciones o actividades escolares o para escolares? | | | | |
| --- | --- | --- | --- | --- |
| **Nunca** | **Rara Vez** | **A veces** | **A menudo** | **Todos los días** |
| 1. ¿Con que frecuencia tú o tus compañeros de clase **amenazan** a alguien para que haga cosas que le dañan, **ofenden** o que no desea hacer? | | | | |
| **Nunca** | **Rara Vez** | **A veces** | **A menudo** | **Todos los días** |
| 1. ¿Con qué frecuencia tu o cualquiera de tus compañeros de clase se **burlan** de alguien, lo **insultan**, **castigan**, **golpean**, o **hieren**? | | | | |
| **Nunca** | **Rara Vez** | **A veces** | **A menudo** | **Todos los días** |
| 1. ¿Con qué frecuencia tu o alguno de tus compañeros de clase **culpan** o **acusan** a alguien de un delito que puede o no haber comprometido? | | | | |
| **Nunca** | **Rara Vez** | **A veces** | **A menudo** | **Todos los días** |
| 1. ¿En las últimas cuatro semanas, ¿cuántas veces has tenido **dolor de estómago**,   **de cabeza**, **pérdida de apetito** o **problemas para dormir**? | | | | |
| **Nunca** | **Rara Vez** | **A veces** | **A menudo** | **Todos los días** |
