# Supplementary material for: Design and validation of a self-administered test to assess bullying (bull-M) in high school Mexicans: a pilot study
Source: BMC Public Health. 2013 Apr 11;13:334. doi: 10.1186/1471-2458-13-334 (PMC3671223; doi:10.1186/1471-2458-13-334)
Supplement: Additional file 2 — Test on the presence of bullying in and out school (Bull-M). [file 1471-2458-13-334-S2.doc]

Additional file 2

**Test on the presence of bullying in and out school (Bull-M)**

This questionnaire is confidential and the information will be used only for statistical purposes. In this way, your identity will be protected. We ask you do not put your name anywhere on the document and to answer each question as honest as possible about what happens in or out school, either by you, your classmates or friends. Furthermore, mark with an “**X”** the actions (**in bold**) which most apply to you or what you do. Example:


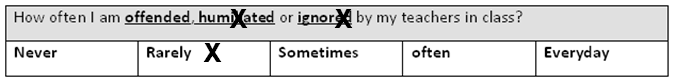


This means that you are rarely humiliated or ignored by your teachers in class

| Age: y Gender: Male ( ) Female ( ) |
| --- |

What happens in or out school?

| 1. How often do your classmates allow or invite you to participate in their games, school activities or extracurricular activities? | | | | |
| --- | --- | --- | --- | --- |
| **Never** | **Rarely** | **Sometimes** | **Often** | **Everyday** |
| 1. How often are you **excluded**, **rejected**, or **expelled** by your classmates in games, conversations, school activities or extracurricular activities? | | | | |
| **Never** | **Rarely** | **Sometimes** | **Often** | **Everyday** |
| 1. How often are you **forced** or **threatened** by your classmates to do things that hurt you, offend you, or that you do not wish to do? | | | | |
| **Never** | **Rarely** | **Sometimes** | **Often** | **Everyday** |
| 1. How often have your classmates **made fun** of you, **insulted** you, **punish** you, **hit** you, or **hurt** you? | | | | |
| **Never** | **Rarely** | **Sometimes** | **Often** | **Everyday** |
| 1. How often are **blamed** or **accused** by your classmates for any offense that may or may not have made you? | | | | |
| **Never** | **Rarely** | **Sometimes** | **Often** | **Everyday** |

What do you or your classmates do in or out school?

| 1. How many times have you or your classmates excluded, rejected or expelled someone from games, conversations or school activities? | | | | |
| --- | --- | --- | --- | --- |
| **Never** | **Rarely** | **Sometimes** | **Often** | **Everyday** |
| 1. How often do you or any of your classmates **threaten** someone to do things that may damage him or her, that **offend** him or her or do not wish to do? | | | | |
| **Never** | **Rarely** | **Sometimes** | **Often** | **Everyday** |
| 1. How often do you or any of your classmates **make fun**, **insult**, **punish**, **hit** or **hurt** of someone? | | | | |
| **Never** | **Rarely** | **Sometimes** | **Often** | **Everyday** |
| 1. How often do you or any of your classmates **blame** or **accuse** someone else from any offense that may or may not have committed? | | | | |
| **Never** | **Rarely** | **Sometimes** | **Often** | **Everyday** |
| 1. In the last four weeks, how often have you had a **stomach ache**, **headache**,   **loss of appetite** or **problems to sleep**? | | | | |
| **Never** | **Rarely** | **Sometimes** | **Often** | **Everyday** |
