# Supplementary material for: Design and validation of a self-administered test to assess bullying (bull-M) in high school Mexicans: a pilot study
Source: BMC Public Health. 2013 Apr 11;13:334. doi: 10.1186/1471-2458-13-334 (PMC3671223; doi:10.1186/1471-2458-13-334)
Supplement: Additional file 3 — Expert judgment validity test (EJVT). [file 1471-2458-13-334-S3.doc]

Additional file 3

**Expert judgment validity test (EJVT)**

Please first read the *Test on the presence of bullying in and out school*. Then, please answer the following questions.

1. Considering that the purpose of the questionnaire is to find whether there is bullying in schools, to what extent you think the questions define the purpose?

|  | **Poorly** |  | **Fairly** |  | **Sufficiently** |
| --- | --- | --- | --- | --- | --- |

1. To what extent you think the questionnaire is clear?

|  | **Poorly** |  | **Fairly** |  | **Sufficiently** |
| --- | --- | --- | --- | --- | --- |

1. To what extent do you think each question is useful for the purpose set out by the questionnaire?

|  | **Poorly** |  | **Fairly** |  | **Sufficiently** |
| --- | --- | --- | --- | --- | --- |

1. To what extent do you think the questionnaire (in both, introductory explanation and survey sections) presents the information necessary for respondents (elementary-to-high school students) in order to answer truthfully?.

|  | **Poorly** |  | **Fairly** |  | **Sufficiently** |
| --- | --- | --- | --- | --- | --- |

1. To what extent do you think the items on the questionnaire sufficiently cover the central (construct) idea?

|  | **Poorly** |  | **Fairly** |  | **Sufficiently** |
| --- | --- | --- | --- | --- | --- |

1. To what extent do you think each question is mutually exclusive from each other (not redundant)?

|  | **Poorly** |  | **Fairly** |  | **Sufficiently** |
| --- | --- | --- | --- | --- | --- |

1. To which extent do you think the questionnaire offers enough questions to be answered properly?

|  | **Poorly** |  | **Fairly** |  | **Sufficiently** |
| --- | --- | --- | --- | --- | --- |

1. To what degree do you think the items on the questionnaire are ambiguous?

|  | **Poorly** |  | **Fairly** |  | **Sufficiently** |
| --- | --- | --- | --- | --- | --- |

1. To what extent do you think the items clearly represent all situations of the targeted population?

|  | **Poorly** |  | **Fairly** |  | **Sufficiently** |
| --- | --- | --- | --- | --- | --- |

1. To what extent do you think the items are relevant to fulfill the purpose of the questionnaire?

|  | **Poorly** |  | **Fairly** |  | **Sufficiently** |
| --- | --- | --- | --- | --- | --- |

1. To what extent do you think the items may be intimidating for answering truthfully?

|  | **Poorly** |  | **Fairly** |  | **Sufficiently** |
| --- | --- | --- | --- | --- | --- |

1. To what extent do you think the questionnaire protects the anonymity of the respondents?

|  | **Poorly** |  | **Fairly** |  | **Sufficiently** |
| --- | --- | --- | --- | --- | --- |

1. To what extent do you think the way of applying (only the pollster and the respondent are present) the questionnaire protects the anonymity of the respondents?

|  | **Poorly** |  | **Fairly** |  | **Sufficiently** |
| --- | --- | --- | --- | --- | --- |

1. To what extent do you think the sequence of the questions are logically ordered?

|  | **Poorly** |  | **Fairly** |  | **Sufficiently** |
| --- | --- | --- | --- | --- | --- |

1. To what extent do you think the typography of the questionnaire is pleasing to the eye

|  | **Poorly** |  | **Fairly** |  | **Sufficiently** |
| --- | --- | --- | --- | --- | --- |

1. To what extent do you think the presentation of the questionnaire is pleasing to the eye

|  | **Poorly** |  | **Fairly** |  | **Sufficiently** |
| --- | --- | --- | --- | --- | --- |

1. To what extent do you think the questions are presented in an organized way?

|  | **Poorly** |  | **Fairly** |  | **Sufficiently** |
| --- | --- | --- | --- | --- | --- |

1. To what extent do you think the two sections of the questionnaire are logically grouped together?

|  | **Poorly** |  | **Fairly** |  | **Sufficiently** |
| --- | --- | --- | --- | --- | --- |

1. To what extent do you think the possible answers to the questionnaire (never, rarely, sometimes, often, everyday) cover all possible options?

|  | **Poorly** |  | **Fairly** |  | **Sufficiently** |
| --- | --- | --- | --- | --- | --- |
